# The association between soluble ST2 and transplant-free survival in precapillary pulmonary hypertension

**DOI:** 10.1016/j.jhlto.2026.100661

**Published:** 2026-08-12

**Authors:** Håvard Ravnestad, Salaheldin Ahmed, Charlotte M. Østby, Einar Gude, John-Peder Escobar Kvitting, Kaspar Broch, Annika E. Michelsen, Thor Ueland, Göran Rådegran, Arne K. Andreassen

**Affiliations:** a Department of Cardiology, Oslo University Hospital, Rikshospitalet, Oslo, Norway; b Institute of Clinical Medicine, University of Oslo, Oslo, Norway; c Department of Clinical Sciences, Cardiology, Lund University, Lund, Sweden; d The Hemodynamic Lab, The Section for Heart Failure and Valvular Disease, Skåne University Hospital, Lund, Sweden; e Department of Education and Research, Helsingborg Hospital, Helsingborg, Sweden; f Department of Cardiothoracic Surgery, Oslo University Hospital, Rikshospitalet, Oslo, Norway; g Research Institute of Internal Medicine, Oslo University Hospital, Rikshospitalet, Oslo, Norway; h Thrombosis Research Center (TREC), Division of Internal Medicine, University Hospital of North Norway, Tromsø, Norway

**Keywords:** PAH, CTEPH, transplant-free survival, soluble ST2, precapillary pulmonary hypertension

## Abstract

**Background:**

Soluble ST2 (sST2) is a decoy receptor for interleukin-33 and a prognostic biomarker in heart failure, but its utility in precapillary pulmonary hypertension (PH) is not well established. Therefore, we aimed to evaluate the prognostic value of sST2 in patients with pulmonary arterial hypertension (PAH) and chronic thromboembolic pulmonary hypertension (CTEPH) at diagnosis and follow-up.

**Materials and methods:**

In this prospective observational cohort study, we measured plasma sST2 by sandwich enzyme-linked immunosorbent assay in samples taken at diagnostic right heart catheterization from 202 treatment naïve Norwegian patients with precapillary PH (n = 115 with PAH, n = 87 with CTEPH). In an external validation cohort, we included 185 treatment naïve Swedish patients with precapillary PH (n = 121 with PAH, n= 64 with CTEPH). In a subset of patients, we measured sST2 levels at follow-up in the Norwegian discovery cohort (n=63) and the Swedish validation cohort (n=121).

**Results:**

On univariable Cox regression, log sST2 was firmly associated with transplant-free survival in the Norwegian discovery cohort (HR 1.92, *P =* 0.005) and in the Swedish validation cohort (HR 2.64, *P* <.001). When adjusting for age and NT-proBNP, sST2 levels were no longer significantly associated with transplant-free survival in the discovery cohort, while remaining independently associated with the outcome in the validation cohort. The change in sST2 from baseline to follow-up was not associated with transplant-free survival in either cohort.

**Conclusion:**

Plasma sST2 levels were univariably associated with death or lung transplantation in PAH and CTEPH but were no longer prognostic after adjustment for age and NT-proBNP. The change in sST2 from baseline to follow-up did not predict transplant-free survival.

## Introduction

Pulmonary arterial hypertension (PAH) is a rare vasculopathy primarily affecting pulmonary arteries, imposing a significant societal and economic burden globally.^1^ Despite significant advances in treatment options over the past decades, PAH remains a disease with high morbidity and mortality.^2^, ^3^ The 2022 European Society of Cardiology (ESC) / European Respiratory Society (ERS) pulmonary hypertension guidelines emphasize prognostic risk stratification at baseline and follow-up to guide treatment in PAH.^4^ Therefore, prognostic biomarkers play an important role in risk stratification and may support the choice between treatment strategies.^4^ Apart from natriuretic peptides, additional biomarkers may enhance the prognostic accuracy of risk stratification tools.^5^, ^6^

ST2 (Suppression of Tumorigenicity 2) is the receptor for Interleukin-33 and is expressed as a transmembrane receptor (ST2L) and a soluble form (sST2). Interleukin-33 and ST2L are expressed in both cardiomyocytes and cardiac fibroblasts, and the expression of these molecules is amplified following cardiac stress.^7^ In animal models, IL-33/ST2L signaling has been shown to reduce myocardial fibrosis and hypertrophy after myocardial injury.^8^ This cardioprotective effect is inhibited by sST2.^9^

Soluble ST2 is a well-established prognostic biomarker in heart failure, and is a strong predictor of outcome in both acute and chronic heart failure, independent of N-terminal pro B-type natriuretic peptide (NT-proBNP).^10^, ^11^, ^12^ This has prompted interest in its potential as a biomarker for conditions primarily affecting the right heart. Apart from left heart failure, studies have demonstrated that sST2 is associated with disease severity in arrhythmogenic cardiomyopathy,^13^ and with disease severity and survival in pulmonary hypertension (PH). 14, 15, 16 However, these studies were either small or retrospective, and the prognostic value of changes in sST2 from baseline to follow-up remains unexplored.

Chronic thromboembolic pulmonary hypertension (CTEPH) is a distinct entity among patients with precapillary PH. While its pathogenesis differs from that of PAH, the microvascular pathology observed in CTEPH closely resemble that seen in PAH.^17^ To date, only a few studies have investigated the utility of sST2 in patients with CTEPH.^15^, ^18^

In light of this, we aimed to examine the association between sST2 at the time of PH diagnosis and survival free from lung transplantation in patients with PAH and CTEPH, and whether the change in sST2 from baseline to follow-up is associated with survival.

## Methods

### Patient population

In this prospective observational cohort study, we included patients diagnosed with PAH or CTEPH at Oslo University Hospital, Rikshospitalet between October 1998 and December 2024. For the validation cohort, we included patients with PAH or CTEPH prospectively enrolled in Lund Cardiopulmonary Registry (LCPR) between September 2011 and January 2024. All patients provided written informed consent. The study was performed in accordance with the Declarations of Helsinki and Istanbul. The Regional Committee for Medical and Health Research Ethics, South-Eastern Norway (2008/10604 and 2017/2128) and the ethics committee at Lund University Hospital (Dnr: 2010/114, 2010/442, 2011/368, 2011/777, 2014/92, and 2015/270) approved the study.

### Diagnosis and right heart catheterization

A diagnosis of PAH or CTEPH was made according to the prevailing diagnostic algorithm at the time of inclusion, most recently the 2022 ESC/ERS Guidelines for the diagnosis and treatment of PH,^4^ and pre-capillary PH was confirmed by right heart catheterization (RHC). RHC was performed predominantly via the right internal jugular vein (or right femoral vein if this was not feasible). We used a Swan-Ganz catheter (Edwards Lifesciences, Irvine, CA) to measure pressures in the right atrium, the right ventricle, the pulmonary artery, and in the pulmonary artery wedge position. Cardiac output was estimated by thermodilution, averaging three to five measurements (five in case of atrial fibrillation). We measured mixed venous oxygen saturation in blood drawn from the pulmonary artery.

### Blood sampling and storage

We collected blood samples in treatment-naïve patients, from the venous introducer during the diagnostic RHC. Ethylenediaminetetraacetic acid was used as anticoagulant. The samples were put on ice immediately after procurement. After centrifugation, plasma was isolated, stored at −80°C and thawed for analysis. Samples from the validation cohort were shipped to Oslo University Hospital, Rikshospitalet on dry ice. All sST2 analyses were done in plasma, and performed at the Research Institute of Internal Medicine, Oslo University Hospital, Rikshospitalet. We measured sST2 using solid phase sandwich enzyme-linked immunosorbent assays from R&D Systems (Human ST2/IL-33R DuoSet ELISA, Bio-Techne, Minneapolis, MI). Hemoglobin, creatinine and NT-proBNP were analyzed as part of routine clinical work-up, at the central labs of Oslo University Hospital, Rikshospitalet, and Skåne University Hospital.

### Statistical analysis

Data are presented as mean ± SD or median (interquartile range) unless otherwise specified. We used Student’s *t*-test to compare normally distributed variables between groups, and Mann-Whitney U-test for non-normally distributed data. For categorical variables, we used Fisher’s exact test or χ^2^ test as appropriate. We analyzed associations between sST2 and other clinical parameters using univariable linear regression. Our prespecified primary endpoint was survival free from lung transplantation. Survival was analyzed using the Cox proportional hazards method, with death or lung transplantation as events (whichever occurred first). NT-proBNP and sST2 were log-transformed before regression analyses. We tested the proportional hazards assumption using Schoenfeld residuals. No imputations were applied; all analyses were performed on complete cases. We assessed model discrimination using Harrell’s C-statistic, reported with 95% confidence intervals in parentheses. We performed all statistical analyses using STATA SE v19.5 (StataCorp, College Station, TX, USA).

## Results

### Patient population

Table 1 shows the baseline characteristics of the two populations. Patients in the Norwegian discovery cohort were younger and less likely to be female compared to patients in the Swedish validation cohort. Comorbidities were similar between the two cohorts. NT-proBNP was considerably higher in the Swedish cohort. Cardiac index and mixed venous oxygen saturation were lower in the Swedish cohort, while there was no difference in mean pulmonary artery pressure (mPAP) or pulmonary vascular resistance (PVR).

**Table 1:** Baseline Characteristics

**Demographic variables**	**Norwegian cohort**	**Swedish cohort**	***P*****value**
Patients	202	185	
Age	56±16	67±15	<.001
Female, n (%)	125 (62)	133 (72)	.04
Body mass index, kg/m^2^	27.5±6.0	26.8±5.3	.59
PH classification			
PAH, n (%)	115 (57)	121 (65)	.088
IPAH	35 (30)	64 (53)	-
HPAH	3 (3)	4 (3)	-
Toxins	4 (4)	0	-
APAH	68 (59)	53 (44)	-
PVOD	2 (2)	0	-
Other	3 (3)	0	-
CTEPH, n (%)	87 (43)	64 (35)	.088
Comorbidity			
Smokers, current or previous, n (%)	72 (44)	N/A	-
Hypertension, n (%)	94 (47)	62 (34)	.31
Diabetes mellitus, n (%)	20 (10)	18 (9)	.50
Coronary heart disease, n (%)	14 (7)	21 (11)	.38
COPD or emphysema, n (%)	11 (5)	8 (4)	.65
Cancer, n (%)	20 (10)	N/A	-
History of VTE, n (%)	62 (202)	N/A	-
Thyroid disease, n (%)	26 (13)	20 (11)	.21
Atrial fibrillation, n (%)	17 (8)	17 (9)	.86
Physical capacity			
Peak VO2, % of exp.	50±29	N/A	-
6MWT (m)	N/A	291±133	-
NYHA class, % (I/II/III/IV)	2/26/64/9	1/30/68/2	.16
Clinical biomarkers			
Creatinine (µmol/L)	91±44	89±30	.76
NT-proBNP (ng/L)	491 (135–1893)	1384 (355–2958)	<.001
Hemoglobin (g/dL)	13.6±3.9	13.9±2.1	.55
Spirometry			
FVC, % of exp.	88±21	N/A	-
FEV1, % of exp.	78±19	N/A	-
DLCO, % of exp.	57±20	54±24	.51
Hemodynamic variables			
RAP, mmHg	7±4	7±5	.39
mPAP, mmHg	45±13	44±12	.60
PAWP, mmHg	9±7	9±4	.40
Cardiac index, L/min/m^2^	2.5±0.8	2.3±0.6	.002
PVR, Wood units	8.7±4.9	9.1±4.6	.35
SvO2, %	63±10	61±10	.01

Data are mean±SD or median (IQR) unless otherwise stated. PH: pulmonary hypertension. PAH: pulmonary arterial hypertension. IPAH: idiopathic pulmonary arterial hypertension. HPAH: hereditary pulmonary arterial hypertension. APAH: associated pulmonary arterial hypertension. PVOD: pulmonary veno-occlusive disease. CTEPH: chronic thromboembolic pulmonary hypertension. COPD: chronic obstructive pulmonary disease. VTE: venous thromboembolism. 6MWT: six-minute walk test. NYHA: New York Heart Association. FVC: forced vital capacity. FEV1: forced expiratory volume during first second. DLCO: diffusion capacity for carbon monoxide. RAP: right atrial pressure. mPAP: mean pulmonary artery pressure. PAWP: pulmonary artery wedge pressure. PVR: pulmonary vascular resistance. SvO2: mixed venous oxygen saturation.

### Soluble ST2 levels in the Norwegian discovery cohort

Plasma sST2 levels are shown in Table 2. There was no difference in sST2 between the PAH and CTEPH patients (*P =*.90). Soluble ST2 levels were higher in patients who died or were lung transplanted compared to transplant-free survivors. This difference was apparent in both the PAH subgroup and in the CTEPH subgroup.

**Table 2:** Plasma Soluble ST2 Levels (ng/mL)

	**Norwegian discovery cohort**			
	**All pts.**	**Survivors**^a^	**Died / LTx**^b^	***P*****value**^c^
Total cohort	47 (34−62)	43 (33−55)	54 (41−72)	.001
PAH	46 (36−63)	42 (33−53)	55 (41−80)	.01
CTEPH	46 (35−62)	44 (34−59)	52 (42−67)	.04
	**Swedish validation cohort**			
	**All pts.**	**Survivors**^a^	**Died / LTx**^b^	***P*****value**
Total cohort	72 (55−93)	66 (48−86)	76 (57−108)	.005
PAH	77 (57−99)	70 (47−88)	82 (58−110)	.01
CTEPH	63 (51−78)	57 (52−76)	67 (48−78)	.58

Plasma levels of soluble ST2. Data are presented as median (IQR) and given in ng/mL.

a Transplant-free survivors.

b Patients who died or were lung transplanted

c P-value for difference between transplant-free survivors and patients who died or were lung transplanted.

Follow-up blood samples were available for 44 patients with PAH and 19 patients with CTEPH. The median time from baseline to follow-up was 3 (2−5) months for PAH patients and 9 (8−11) months for CTEPH patients. The mean difference in sST2 was −4±24 ng/mL in PAH (*P =*.08) and −6±27 ng/mL in CTEPH (*P =*.39).

On univariable linear regression, we found weak correlation between log sST2 and mPAP in CTEPH patients (R = 0.33, *P =*.002), but no correlation in PAH patients (R = 0.008, *P =*.42). There was no correlation between the duration of sample storage and sST2 levels (R = 0.07, *P =*.33).

### Soluble ST2 levels in the Swedish validation cohort

**Figure 1:**
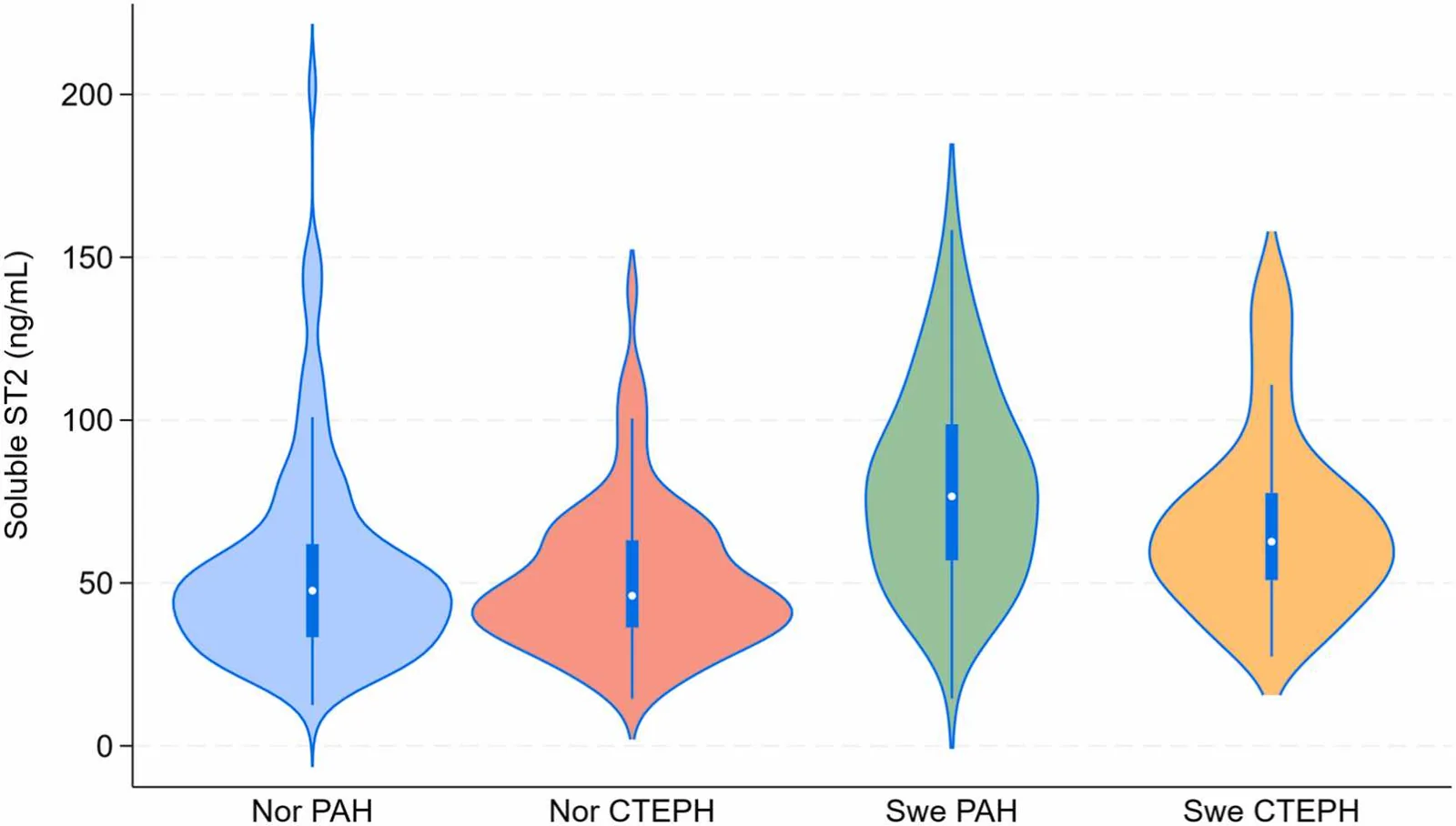
Violin-plot showing the distribution of soluble ST2-levels in the Norwegian (Nor) discovery and Swedish (Swe) validation cohorts according to diagnosis groups (non-transformed data). PAH: pulmonary arterial hypertension. CTEPH: chronic thromboembolic pulmonary hypertension.

Plasma sST2 was higher in PAH patients than in CTEPH patients (*P =*.006). As in the Norwegian cohort, we found higher sST2 levels in patients who died or were lung transplanted compared to transplant-free survivors. This difference was also apparent in patients with PAH, but not in patients with CTEPH. Figure 1 shows a violin plot of sST2-levels in the Norwegian and Swedish PAH and CTEPH patients.

Follow-up blood samples were available in 121 individuals, all of whom had PAH. The median time from baseline to follow-up was 3 (3−5) months. The mean difference in sST2 between baseline and follow-up was −7±23 ng/mL (*P =*.001).

**Figure 2:**
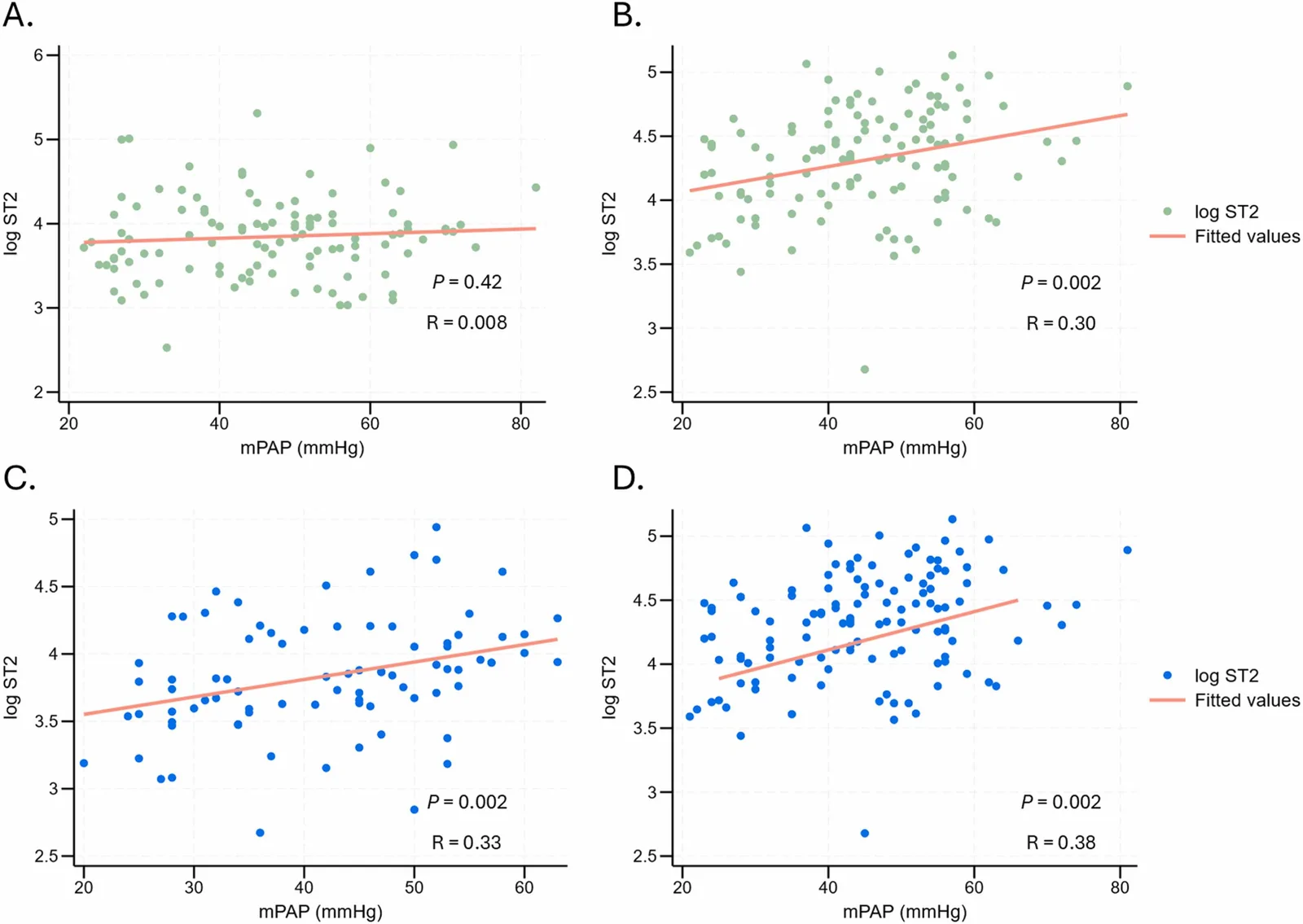
Scatter plots of log soluble ST2 versus mean pulmonary artery pressure (mPAP), with red line representing fitted values. Upper panels show Norwegian (A) and Swedish (B) PAH patients. Lower panels show Norwegian (C) and Swedish (D) CTEPH patients.

On univariable linear regression, we found weak correlations between log sST2 and mPAP at baseline in PAH (R=0.30, *P =*.002) as well as in CTEPH (R = 0.38, *P =*.002). There was no correlation between the duration of sample storage and plasma sST2 levels (R = 0.08, *P =*.26). Figure 2 shows a scatter plot of log sST2 and mPAP in the Norwegian and the Swedish cohorts.

### Survival and lung transplantation, Norwegian cohort

The median observation time was 37 months (15−67). During the study period, 71 patients died or were lung transplanted. Among these, 47 had PAH and 24 had CTEPH. Eight patients were transplanted, seven of these had PAH and one had CTEPH. On univariate Cox regression, log sST2 was firmly associated with death or lung transplantation (HR 1.92, *P =*.005). However, after adjusting for age and NT-proBNP in multivariate Cox regression, log sST2 was no longer significantly associated with transplant-free survival (*P =*.97), whereas NT-proBNP (*P* <.001) remained associated with the outcome. Assessed by Harrel’s C-statistic, log sST2 predicted transplant-free survival with an accuracy of 0.62 (95% CI, 0.54–0.70) and 0.76 (95% CI, 0.68–0.82) in the univariate and multivariate models respectively. Looking at the PAH and CTEPH populations separately, log NT-proBNP remained associated with death or lung transplantation but sST2 did not. The change in sST2 from baseline to follow-up was not associated with transplant-free survival (*P =*.18).

### Survival and lung transplantation, Swedish cohort

**Figure 3:**
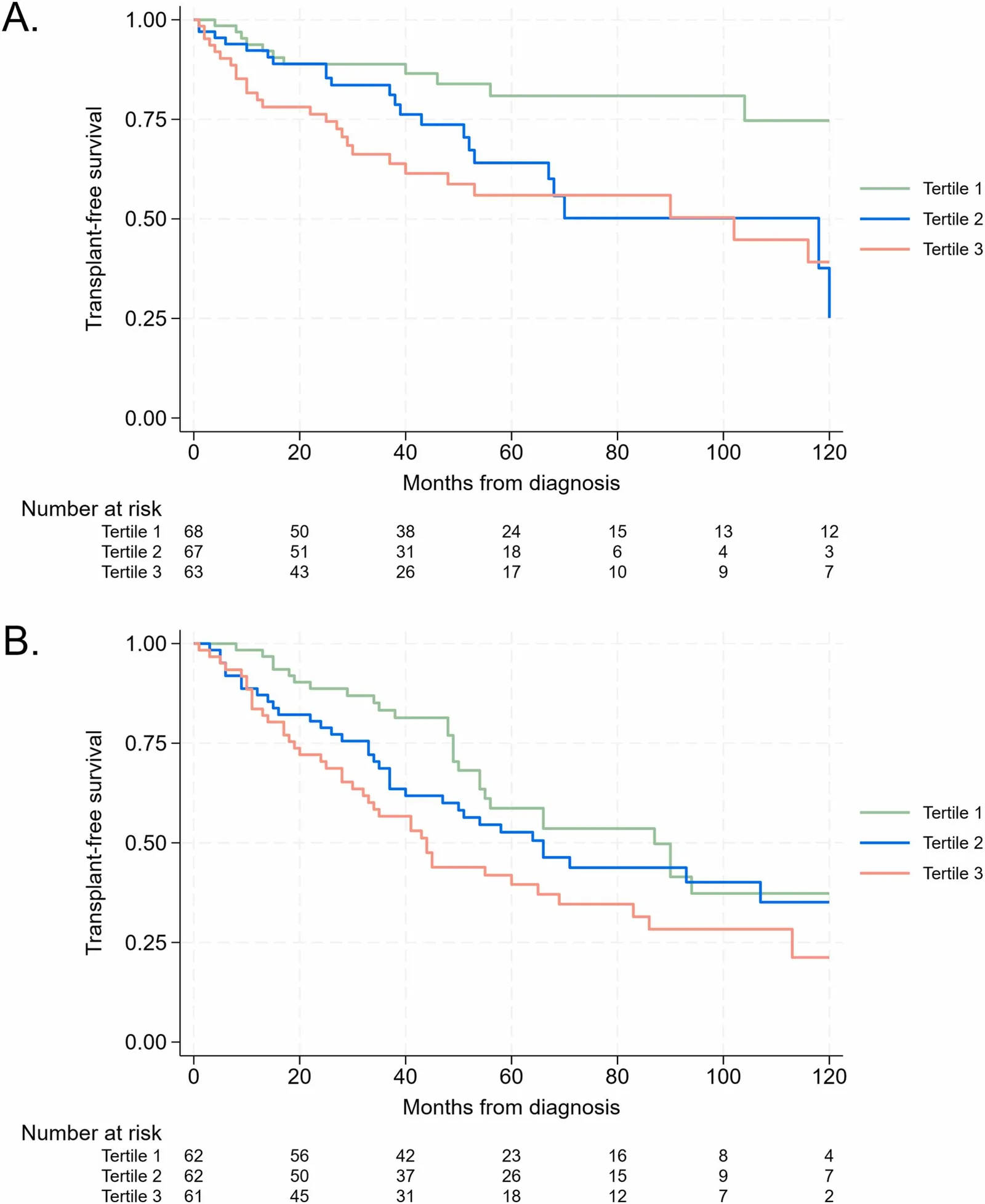
A: Kaplan-Meier plot of transplant-free survival according to soluble ST2 tertiles (non-transformed values) for the Norwegian cohort. B: Kaplan-Meier plot of transplant-free survival according to soluble ST2 tertiles (non-transformed values) for the Swedish cohort.

The median observation time was 48 months (26−73). A total of 104 patients died or had lung transplantation during the study period. Of these, 74 were PAH patients and 30 were CTEPH patients. Five patients had lung transplantation, all of them for PAH. Table 3 shows survival according to diagnosis group and sST2 levels in both Norwegian and Swedish cohorts. Figure 3 shows Kaplan-Meier plots of transplant-free survival according to sST2-tertiles.

**Table 3:** Transplant-free Survival

	**Norwegian cohort**						**Swedish cohort**					
	**n**	**1 year**	**n**	**2 years**	**n**	**10 years**	**n**	**1 year**	**n**	**3 years**	**n**	**10 years**
All patients	160	89 (83−93)	105	80 (73−85)	22	49 (37−61)	167	89 (84−93)	121	69 (61−75)	13	32 (23−40)
PAH	94	89 (81−94)	61	76 (67−84)	13	40 (26−55)	108	88 (80−92)	74	64 (54−72)	7	27 (17−38)
CTEPH	67	89 (80−94)	44	85 (74−91)	10	67 (51−79)	60	92 (82−97)	47	78 (66−86)	8	39 (23−55)
sST2, 1st tertile	59	94 (84−98)	42	88 (78−95)	13	75 (55−87)	62	98 (89−100)	46	82 (69−89)	5	37 (21−53)
sST2, 2nd tertile	56	92 (82−97)	36	84 (72−91)	3	25 (5−52)	55	85 (74−92)	41	67 (54−78)	7	34 (20−49)
sST2, 3rd tertile	46	80 (67−88)	29	66 (52−77)	8	39 (21−57)	54	84 (72−91)	34	57 (43−68)	3	21 (8−38)

Survival at 1, 3 and 10 years according to diagnosis group and soluble ST2 (sST2) level tertiles for the Norwegian and Swedish cohorts, with 95% confidence intervals in parentheses. PAH: pulmonary arterial hypertension. CTEPH: chronic thromboembolic pulmonary hypertension.

As in the Norwegian cohort, log sST2 was robustly associated with death or lung transplantation on univariate Cox regression (HR 2.64, *P* <.001). After adjustment for age and log NT-proBNP, log ST2 remained independently associated with this outcome (*P =*.001). Using Harrel’s C-statistic, log sST2 predicted transplant-free survival with an accuracy of 0.62 (95% CI, 0.56–0.67) in the univariate model and 0.73 (95% CI, 0.68–0.79) for the multivariate model. Table 4 summarizes results from univariate and multivariate Cox regression from both Norwegian and Swedish cohorts. The change in sST2 from baseline to follow-up was not associated with transplant-free survival (*P =*.29).

**Table 4:** Cox Regression

	**Norwegian cohort**										**Swedish cohort**									
		**Univariate**					**Multivariate**					**Univariate**					**Multivariate**			
**All patients**	**n**	**HR**	**z**	***P*****value**	**95% CI**		**HR**	**z**	***P*****value**	**95% CI**	**n**	**HR**	**z**	***P*****value**	**95% CI**		**HR**	**z**	***P*****value**	**95% CI**
Age	202	1.01	1.2	.09	1.00–1.03		1.01	1.2	.22	0.99–1.03	185	1.06	5.1	<.001	1.03–1.08		1.06	5.1	<.001	1.04–1.09
log NT-proBNP	167	1.79	4.5	<.001	1.39–2.30		1.78	3.6	<.001	1.39–2.32	180	1.52	5.2	<.001	1.30–1.78		1.23	2.3	.02	1.03–1.47
log sST2	202	1.92	2.8	.005	1.22–3.00		0.55	0.0	.97	0.49–2.11	185	2.64	3.5	<.001	1.55–4.53		2.84	3.2	.001	1.49–5.39
DLCO, % of exp.	131	0.96	-4.1	<.001	0.95–0.98		-	-	-	-	82	0.98	-3.2	<.001	0.96–0.99		-	-	-	-
PAH patients																				
Age	115	1.01	0.3	.26	0.99–1.03		1.01	0.7	.46	0.97–1.04	121	1.05	4.5	<.001	1.03–1.07		1.05	4.0	<.001	1.03–1.08
log NT-proBNP	94	1.74	3.3	.001	1.25–2.42		1.78	3.2	.001	1.25–2.52	118	1.53	4.3	<.001	1.26–1.86		1.30	2.4	.02	1.05–1.63
log sST2	115	1.60	1.7	.09	0.92–2.77		0.83	-0.4	.71	0.31–2.23	121	2.33	2.6	.01	1.22–4.44		1.87	1.6	.12	0.85–4.11
DLCO, % of exp.	78	0.96	-3.5	<.001	0.94–0.98		-	-	-	-	61	0.98	-2.5	.01	0.96–0.99		-	-	-	-
CTEPH patients																				
Age	87	1.03	1.9	.05	1.0–1.07		1.01	0.6	.57	0.97–1.05	64	1.11	3.5	<.001	1.05–1.19		1.13	3.6	<.001	1.06–1.21
log NT-proBNP	73	2.07	3.2	.001	1.32–3.25		2.00	3.0	.003	1.26–3.16	62	1.46	1.6	.008	1.10–1.92		0.47	0.7	.47	0.82–1.53
log sST2	87	3.14	2.4	.02	1.25–7.90		1.25	0.4	.70	0.39–3.99	64	2.38	1.6	.11	0.82–6.96		3.6	2.0	.04	1.05–12.6
DLCO, % of exp.	53	0.97	-2.0	.05	0.93–1.00		-	-	-	-	21	1.00	0.0	.99	0.97–1.03		-	-	-	-

Univariate and multivariate Cox regression of survival free from lung transplantation. z = Wald statistic, computed as $z=\hat{\beta }∕\mathrm{SE}\left(\hat{\beta }\right)$. PAH: pulmonary arterial hypertension. CTEPH: chronic thromboembolic pulmonary hypertension. NT-proBNP: N-terminal pro-B-type natriuretic peptide. sST2: soluble ST2. DLCO: diffusion capacity for carbon monoxide.

### Survival and DLCO

We performed a secondary multivariate Cox regression including age, log NT-proBNP, log sST2 and DLCO. In the discovery cohort, DLCO (*P =*.01) and log NT-proBNP (*P =*.001) were independently associated with transplant-free survival (see Table S1 in the online supplement) but not log sST2. In the Swedish validation cohort, only age remained associated with the outcome.

## Discussion

In this prospective observational cohort study, we have shown that sST2 is elevated in patients with precapillary PH. The levels were higher in subjects who died or were lung transplanted compared to transplant-free survivors. Higher levels of sST2 were associated with increased risk of death or lung transplantation. In the Norwegian discovery cohort, the predictive value of sST2 was negated when adding age and log NT-proBNP to the regression model. This was true in all patients, and in PAH and CTEPH separately. In the Swedish validation cohort, log sST2 remained independently associated with transplant-free survival in the multivariate analysis, but correlated weakly with PA pressures. The change in sST2 from baseline to follow-up was not associated with transplant-free survival.

To our knowledge, this is the second largest study on sST2 in patients with precapillary PH, the first to include an external validation cohort, and the largest prospective study to date. We are also the first to describe the predictive value of change in sST2 from baseline to follow-up.

Soluble ST2 levels were higher in the Swedish validation cohort, compared to the Norwegian cohort. This may be in part due to older and more severely ill patients in the Swedish cohort, as suggested by the considerably higher NT-proBNP levels and higher mortality in the Swedish patients compared with the Norwegian patients. Despite the higher NT-proBNP levels in the Swedish validation cohort, the association between NT-proBNP and transplant-free survival was stronger in the Norwegian discovery cohort.

ST2 is a receptor in the interleukin (IL)-1 receptor family, expressed as a transmembrane receptor (ST2L), and as a soluble form (sST2). The ligand of ST2 is interleukin-33 (IL-33), a nuclear alarmin.^19^, ^20^ Signaling through the IL-33/ST2L pathway plays an important role in Type 2 immune responses,^20^ but has also been shown to facilitate wound healing and promote organ fibrosis.^21^ The soluble isoform of ST2 acts as a decoy receptor for IL-33, neutralizing IL-33 and thereby modulating the inflammatory response.^7^ The role of the IL-33/ST2 axis in the pathogenesis of PH is incompletely understood, but experimental studies provide some insight. Prolonged IL-33 exposure increases PA pressures in mice,^22^ and leads to PA and RV hypertrophy.^23^ Soluble ST2 levels are elevated in mice with long-term IL-33 exposure.^23^ Hypoxia-induced PH is significantly less pronounced in ST2 knockout mice compared with wild type mice.^24^ Expression of hypoxia inducible factor 1-α (HIF1-α) and vascular endothelial growth factor A (VEGF-A) increases in wild type mice exposed to hypoxia, but not in ST2 knockout mice.^22^ In a cellular model, IL-33 signaling lead to increased expression of HIF1-α and VEGF-A by human pulmonary artery endothelial cells, an effect canceled by knockdown of the ST2 gene.^24^

The pulmonary vascular bed, cardiac fibroblasts and cardiomyocytes are both likely sources of the increased levels of sST2 in patients with precapillary PH. The degree to which each of these contribute is unknown. Soluble ST2 is well documented as a prognostic biomarker in left sided heart failure,^25^, ^26^, ^27^, ^28^, ^29^ although pulmonary sST2 production is a relevant contributor in these patients in addition to sST2 released from the myocardium.^30^ In fact, there are studies suggesting that the elevated ST2 levels in patients with heart failure reflect release from the vascular endothelium.^31^ In PAH, sST2 levels have been shown to correlate with the dimensions, function, and degree of fibrosis of the RV.^14^, ^32^ How much sST2 is produced in the failing RV in the absence of pressure overload is not known. Although studies on the prognostic value of sST2 in primary right heart failure are lacking, we have previously shown that sST2 levels correlate with disease severity in patients with arrhythmogenic right ventricular cardiomyopathy.^13^ In this study, we found only weak correlations between sST2 and PA pressures. In the discovery cohort, sST2 was no longer independently associated with transplant-free survival after adjusting for NT-proBNP. This mirrors the findings reported by Geenen and colleagues in 104 patients with PH of different etiologies.^15^ In a large study on 2.017 PAH patients by Simpson *et al.*, the multivariate Cox regression on the prognostic value of sST2 did not include NT-proBNP.^16^ However, the addition of sST2 to survival models including NT-proBNP (including the REVEAL score) resulted in significant improvements in model fit and prognostic capacity. Right heart failure is the leading cause of death in PAH and CTEPH.^33^, ^34^, ^35^ Hence, the lack of independent association between sST2 and transplant-free survival in the discovery cohort after adjusting for age and NT-proBNP may be a reflection of the high cardiac specificity of NT-proBNP. In the Swedish validation cohort, sST2 remained independently associated with the primary outcome on multivariate analysis. However, the low C-statistic indicates that sST2 has limited value on its own as a biomarker in this population. Additionally, we found no correlation between the change in sST2 from baseline to follow-up and transplant-free survival, suggesting that serial sST2 measurements for prognostication may not be helpful.

In the Norwegian discovery cohort, DLCO was independently associated with transplant-free survival in PAH as well as in CTEPH. While there seems to be a similar trend in the Swedish data, results did not reach statistical significance, although the high proportion of patients with missing data for DLCO in the validation cohort suggests cautious interpretation. DLCO is typically low in patients with PAH associated with connective tissue disease but usually normal or only mildly reduced in IPAH. However, survival is poor in a subset of IPAH patients with severe reduction in DLCO and a history of smoking.^36^ In CTEPH, DLCO has been suggested as a biomarker of microvasculopathy.^37^

This study has several limitations. First, it is observational, and as such causality cannot be assessed. Second, although this is the largest prospective study on sST2 in precapillary PH to date, the relatively small sample size still limits generalizability of our findings. We did not have statistical power to assess potential differences across subtypes of PAH. Third, the Norwegian data did not include 6-minute walk test, instead using cardiopulmonary exercise test to assess physical capacity. This complicates analysis of the effect of adding sST2 to existing risk scoring tools. Fourth, we did not have accurate information on treatment (medical, surgical or interventional) and how the treatment regimens changed over time. Differences in treatment between the cohorts may have influenced survival. We cannot exclude the possibility that differences in prevalence of current or previous smoking may have had an effect on the observed differences between the cohorts, as information on smoking status was lacking in the Swedish validation cohort. Finally, the inclusion period in the Norwegian cohort was very long, and substantial changes in treatment regimen especially for PAH patients during this period might have introduced bias. Due to the study design, we could not adjust for treatment effects.

## Conclusion

In this prospective observational study, sST2 was associated with death or lung transplantation in patients with PAH or CTEPH. However, the change from baseline to follow-up in sST2 was not associated with survival. The predictive capacity of sST2 was no longer apparent in the discovery cohort after adjusting for age and NT-proBNP. Our findings were uniform across PAH and CTEPH patients but differed between the Norwegian discovery and Swedish validation cohorts. In summary, our findings suggest that sST2 has limited prognostic value in precapillary PH on top of existing biomarkers. The results from our study do not support adding sST2 to existing risk scoring tools. New biomarkers should be a primary focus for future research in precapillary PH.

## Financial disclosures

This study received an unrestricted research grant from Alexion Pharmaceuticals, from Nordic Infucare AB and Actelion Pharmaceuticals Ltd. The funders had no role in the planning of this study, data collection and analysis or preparation of the manuscript.

## Declaration of Competing Interest

The authors declare the following financial interests/personal relationships which may be considered as potential competing interests: HR has reveived consulting fees from MSD. AKA has received lecture fees from MSD, Nordic InfuCare and Johnson&Johnson, and has Advisory Board participation for MSD.

## Footnotes

Appendix A Supplemental data associated with this article can be found in the online version at doi:10.1016/j.jhlto.2026.100661.
