# Supplementary material for: The association between soluble ST2 and transplant-free survival in precapillary pulmonary hypertension
Source: JHLT Open. 2026 Aug 12;14:100661. doi: 10.1016/j.jhlto.2026.100661 (PMC13542606; doi:10.1016/j.jhlto.2026.100661)
Supplement: Supplementary file 1 [file mmc1.docx]

| **Table S1.** Cox regression including DLCO (online supplement) | | | | | | | |  |  |  |  |  |  |  |  |  |  |  |  |  |  |
| --- | --- | --- | --- | --- | --- | --- | --- | --- | --- | --- | --- | --- | --- | --- | --- | --- | --- | --- | --- | --- | --- |
|  |  |  |  |  |  |  |  |  |  |  |  |  |  |  |  |  |  |  |  |  |  |
|  | **[Removed, discovery] cohort** | | | | | | | | | |  | **[Removed, validation] cohort** | | | | | | | | | |
|  |  | **Univariate** | | | |  | **Multivariate** | | | |  |  | **Univariate** | | | |  | **Multivariate** | | | |
| **All patients** | **n** | **HR** | **z** | ***P* value** | **95% CI** |  | **HR** | **z** | ***P* value** | **95% CI** |  | **n** | **HR** | **z** | ***P* value** | **95% CI** |  | **HR** | **z** | ***P* value** | **95% CI** |
| Age | 202 | 1.01 | 1.7 | .09 | 1.00 - 1.03 |  | 1.01 | 1.0 | .32 | 0.99 - 1.04 |  | 185 | 1.06 | 5.1 | < .001 | 1.03 - 1.08 |  | 1.03 | 2.3 | .02 | 1.00 - 1.07 |
| log NT-proBNP | 167 | 1.79 | 4.5 | < .001 | 1.39 - 2.30 |  | 1.78 | 3.6 | .001 | 1.31 - 2.43 |  | 180 | 1.52 | 5.2 | < .001 | 1.30 - 1.78 |  | 1.17 | 1.6 | .10 | 0.96 - 1.53 |
| log sST2 | 202 | 1.92 | 2.8 | .005 | 1.22 - 3.00 |  | 0.55 | -1.2 | .24 | 0.20 - 1.50 |  | 185 | 2.64 | 3.5 | < .001 | 1.55 - 4.53 |  | 1.56 | 0.9 | .40 | 0.55 - 4.36 |
| DLCO, % of exp. | 131 | 0.96 | -4.1 | < .001 | 0.95 - 0.98 |  | 0.97 | -2.6 | .01 | 0.95 - 0.99 |  | 82 | 0.98 | -3.2 | < .001 | 0.96 - 0.99 |  | 0.98 | -2.0 | .05 | 0.97 - 1.00 |
|  |  |  |  |  |  |  |  |  |  |  |  |  |  |  |  |  |  |  |  |  |  |
| **PAH patients** |  |  |  |  |  |  |  |  |  |  |  |  |  |  |  |  |  |  |  |  |  |
| Age | 115 | 1.01 | 0.3 | .26 | 0.99 - 1.03 |  | 1.00 | 0.1 | .88 | 0.97 - 1.04 |  | 121 | 1.05 | 4.5 | < .001 | 1.03 - 1.07 |  | 1.02 | 1.3 | .20 | 0.99 - 1.05 |
| log NT-proBNP | 94 | 1.74 | 3.3 | .001 | 1.25 - 2.42 |  | 1.83 | 2.9 | < .001 | 1.21 - 2.78 |  | 118 | 1.53 | 4.3 | < .001 | 1.26 - 1.86 |  | 1.40 | 2.4 | .02 | 1.07 - 1.84 |
| log sST2 | 115 | 1.60 | 1.7 | .09 | 0.92 - 2.77 |  | 0.28 | -1.8 | .08 | 0.07 - 1.16 |  | 121 | 2.33 | 2.6 | .01 | 1.22 - 4.44 |  | 0.94 | -0.1 | .91 | 0.30 - 2.88 |
| DLCO, % of exp. | 78 | 0.96 | -3.5 | < .001 | 0.94 - 0.98 |  | 0.96 | -2.5 | .01 | 0.93 - 0.99 |  | 61 | 0.98 | -2.5 | .01 | 0.96 - 0.99 |  | 0.98 | -1.9 | .06 | 0.97 - 1.00 |
|  |  |  |  |  |  |  |  |  |  |  |  |  |  |  |  |  |  |  |  |  |  |
| **CTEPH patients** |  |  |  |  |  |  |  |  |  |  |  |  |  |  |  |  |  |  |  |  |  |
| Age | 87 | 1.03 | 1.9 | .05 | 1.0 - 1.07 |  | 1.00 | 0.0 | .97 | 0.95 - 1.05 |  | 64 | 1.11 | 3.5 | < .001 | 1.05 - 1.19 |  | 1.13 | 1.9 | .06 | 0.99 - 1.28 |
| log NT-proBNP | 73 | 2.07 | 3.2 | .001 | 1.32 - 3.25 |  | 4.46 | 2.8 | .006 | 1.53 - 13.0 |  | 62 | 1.46 | 1.6 | .008 | 1.10 - 1.92 |  | 0.76 | -1.0 | .34 | 0.43 - 1.34 |
| log sST2 | 87 | 3.14 | 2.4 | .02 | 1.25 - 7.90 |  | 0.73 | -0.4 | .68 | 0.16 - 3.32 |  | 64 | 2.38 | 1.6 | .11 | 0.82 - 6.96 |  | 17.6 | 1.4 | .16 | 0.32 - 977 |
| DLCO, % of exp. | 53 | 0.97 | -2.0 | .05 | 0.93 - 1.00 |  | 0.91 | -2.9 | .004 | 0.85 - 0.97 |  | 21 | 1.00 | 0.0 | .99 | 0.97 - 1.03 |  | 1.00 | 0.1 | .89 | 0.96 - 1.04 |

Univariate and multivariate Cox regression of survival free from lung transplantation. z = Wald statistic, computed as $z=\hat{\beta}/SE\left( \hat{\beta} \right)$. PAH: pulmonary arterial hypertension. CTEPH: chronic thromboembolic pulmonary hypertension. NT-proBNP: N-terminal pro-B-type natriuretic peptide. DLCO: diffusion capacity for carbon monoxide.
